# Erythrocyte Omega-3 Fatty Acid Content in Elite Athletes in Response to Omega-3 Supplementation: A Dose-Response Pilot Study

**DOI:** 10.1155/2017/1472719

**Published:** 2017-06-01

**Authors:** Franchek Drobnic, Félix Rueda, Victoria Pons, Montserrat Banquells, Begoña Cordobilla, Joan Carles Domingo

**Affiliations:** ^1^Departamento de Investigación del CAR, Av. Alcalde Barnils 3, 08173 Sant Cugat del Vallés, Spain; ^2^Servicios Médicos del FC Barcelona, Av. del Sol, s/n, Sant Joan Despí, 08970 Barcelona, Spain; ^3^Departamento de Bioquímica y Biología Molecular, Facultad de Biología, Av. Diagonal 643, 08028 Barcelona, Spain

## Abstract

**Introduction:**

Supplementation of Omega-3 fatty acids (n-3FA) in athletes is related to the anti-inflammatory and/or antioxidant effect and consequently its action on all the processes of tissue restoration and adaptation to physical stress.

**Objective:**

Evaluate the Omega-3 Index (O3Ix) response, in red blood cells, to supplemental EPA + DHA intake in the form of high purity and stable composition gums (G), in elite summer athletes.

**Method:**

Twenty-four summer sport athletes of both sexes, pertaining to the Olympic Training Center in Spain, were randomized to two groups (2G = 760 or 3G = 1140 mg of n-3 FA in Omegafort OKids, Ferrer Intl.) for 4 months. Five athletes and four training staff volunteers were control group.

**Results:**

The O3Ix was lower than 8% in 93.1% of all the athletes. The supplementation worked in a dose-dependent manner: 144% for the 3G dose and 135% for the 2G, both *p* < 0.001, with a 3% significant decrease of Omega-6 FAs. No changes were observed for the control group.

**Conclusions:**

Supplementation with n-3FA increases the content of EPA DHA in the red blood cells at 4 months in a dose-dependent manner. Athletes with lower basal O3Ix were more prone to increment their levels. The study is registered with Protocol Registration and Results System (ClinicalTrials.gov) number NCT02610270.

## 1. Introduction

Docosahexaenoic acid (DHA C22: 6) and eicosapentaenoic acid (EPA C20: 5) are the most important polyunsaturated fatty acids (FAs) known as long-chain Omega-3 (n-3). Both are considered essential FA and are important components of the lipid bilayer of cell membranes. For its incorporation, they should be synthesized from essential fatty acid, alpha-linolenic acid (ALA), or taken directly preformed in the diet. At present, it is suggested that the administration of purified EPA and/or DHA can offer a wide range of beneficial effects [1], ranging from the plasticity, neuronal development, and functionality of the central nervous system [2] to the treatment and prevention of chronic diseases with an inflammatory component [3]. The indication of diet supplementation in sport activities is due to the anti-inflammatory and/or antioxidant effect and consequently its action on all the processes of tissue restoration and adaptation to the physical stress and training, from the connective tissue to the neural development [4]. However, it has been shown that DHA is more effective than EPA in modulating specific markers of inflammation as well as blood lipids [5]. The epidemiological studies related to the impact of the supplementation on the physical activities are focused on supposed actions of the n-3 FA on muscle metabolism and tissue recovery [6], functional performance, and inflammation [7] and, with a very specified indications on sport, as exercise induced asthma [8], traumatic brain injury [9] or injury recovery, training adaptation, and sarcopenia [10]. Those studies report the use of different doses, concentration, and duration, and they do not always reference the previous state of the amount of these FA in cell membranes or usual diet.

The use of biomarker-based approaches has made it possible to study and evaluate with criteria the Omega-3 Index (O3Ix), which is defined as the sum of EPA+DHA content in red blood cell (RBC) membranes and has been considered a risk factor for death from coronary heart disease and as a biomarker of n-3 FA status [11]. An O3Ix of ≥8% has been recommended for its cardiovascular protective effect [12] and has been postulated to be adequate also in the elite athlete [13]. Until now, Von Schacky et al.'s study [13] is the only one that targets elite athlete as reference values for O3Ix. Surprisingly, those subjects, with a diet geared toward better performance, demonstrate not only a low consumption of fish but also a low level O3Ix, far from the desired range over 8%. Well conducted studies have confirmed that dietary or supplemental intake of EPA + DHA is associated with higher levels of the O3Ix [14–16].

In the present study, the objective was to model the O3Ix response to supplemental EPA + DHA intake within attainable dietary ranges in athletes. We had the primary hypothesis that if adherence to treatment is adequate and diet is maintained, a change in the O3Ix would be observed depending on the dosage of the supplementation. This information will be important for making better EPA + DHA recommendations to achieve a target O3Ix for future studies on the evaluation of the effect of n-3 FAs in the different physical exercise activities.

## 2. Methods

This project has tried to follow the guidelines in the design, conduct, and reporting of studies of human health benefits of foods, summarized by Welch et al. [17].

### 2.1. Participants

Eligibility criteria were based on selecting healthy athletes of both sexes, from a specific age range and committed to the project. Athletes belonged to different summer sport federations and lived at the Olympic Training Center (OTC) of Sant Cugat del Vallés (Spain). Exclusion criteria included any type of inflammatory process or the use of anti-inflammatory medications, consumption of n-3 FA supplements and n-3 FA-supplemented foods in the past 3 months. planning to change dietary habits, or training schedule. In order to establish a control group (C), three athletes and four members of the technical staff met the same requirements for maintaining diet, weight, and daily activity level. All of them had their official sport preparticipation screening evaluation, signed informed consent, and went through a medical examination that included medical history, physical examination, anthropometric measurements, complete blood count, and standard chemistry panel to rule out the presence of any newly developed illness or inflammatory process. Diet analysis was conducted to evaluate, through a week registration, all the nutritional components of their diet. The study protocol was approved by the Ethical Committee of the Sports Council of the Generalitat de Catalunya 08/2014/CEIEGC. All procedures followed were in accordance with the ethical standards of the Helsinki Declaration of 1975, revised in 2000.

### 2.2. Intervention

This was a randomized, parallel-group with control subjects study. Participants (*n* = 24), 13 women (55%), were randomized to take two different dosages of n-3 FAs daily, as fish oil supplements in the form of gums (Omegafort OKids, Ferrer Intl.) for 4 months, the approximate time of lifespan of the RBC [17] and time that it takes the composition in FA of cell membranes to reach a new steady state [18]. To ensure even distribution among treatment groups, a computer randomization scheme was used, which is stratified by sex and age and balanced the size of the two blocks. Eligible participants were assigned to treatment group 2G, two gums daily, or 3G, three gums daily (i.e., 2G = 760 mg or 3G = 1140 mg of n-3 FA). All researchers and clinicians, except the Head Dept., and participants, were blinded to treatment assignment. Analysis of the fish oil gums verified that they contained 35,7% DHA, 27,7% EPA, 3.32% docosapentaenoic acid, 18,5% oleic acid, 3,1% vaccenic acid, 1,4% stearic acid, 1,2% palmitic acid, 1,7% arachidonic acid, and small amounts of other fatty acids (Table 1).

All participants were instructed to maintain their training schedule, diet and activity level, and their usual consumption of fatty fish as well as their no consumption of any supplementation during the study course. The participants were contacted monthly to ensure gummies intake compliance and to discuss any difficulties in following the treatment. Also, participants reported back to the Research Department after 8 weeks to return the gum boxes and to receive new supplies.

### 2.3. Blood Sample Collection

Blood samples collection was performed in the fasting state by venipuncture and harvested in K_2_-EDTA-containing tubes before and after the intervention (12 hours without any intake with the exception of water, 48 hours without alcohol, and 12 hours without perform vigorous exercise). After each blood sample collection, a complete blood count and a general biochemistry profile were obtained. Whole blood was centrifuged at 1500 ×g for 15 minutes at 4°C. Except for assays that required unfrozen specimens, samples were stored at −80°C until they were analyzed.

### 2.4. RBC Fatty Acid Analysis

Erythrocytes were separated from the plasma by centrifugation (3000 rpm, 1500 ×g, for 10 min) and washed with an equal volume of saline. These erythrocytes resuspended with saline were stored in a freshly 0.01% butylated hydroxyl toluene- (BHT-) treated Eppendorf vials at −80°C. The fatty acids composition was determined using the method by Lepage and Roy [19]; erythrocyte's membranes were extracted from aliquots of 200 *μ*L of erythrocyte suspensions and the fatty acids converted to methyl esters by reaction with acetyl chloride for 60 min at 100°C. Methyl ester fatty acids (FAME) were separated and analyzed by gas chromatography performed on a Shimadzu GCMS-QP2010 Plus gas chromatograph/mass spectrometer (Shimadzu, Kyoto, Japan) and peaks were identified through mass spectra and by comparing with respect to a reference FAME mixture (GLC-744 Nu-Chek Prep. Inc., Elysian MN, USA) the elution pattern and relative retention times of FAME. The O3Ix was calculated as erythrocyte (EPA + DHA)/(total fatty acids) × 100% (percentage molar of total fatty acids) [11].

### 2.5. Statistical Analysis

Mean changes from baseline to 4 months were calculated and compared between groups using paired* t*-test. Differences among groups were tested by analysis of variance using a general linear model. All statistical tests were performed at a significance level of 0.05. Adjusted *p* < 0.05 was considered significant. Continuous data are reported as the mean ± SD. For descriptive purposes, categorical data are presented as percentages. The statistical software program SPSS for Windows, version 13.0, was used for all data analysis.

## 3. Results

**Figure 1:**
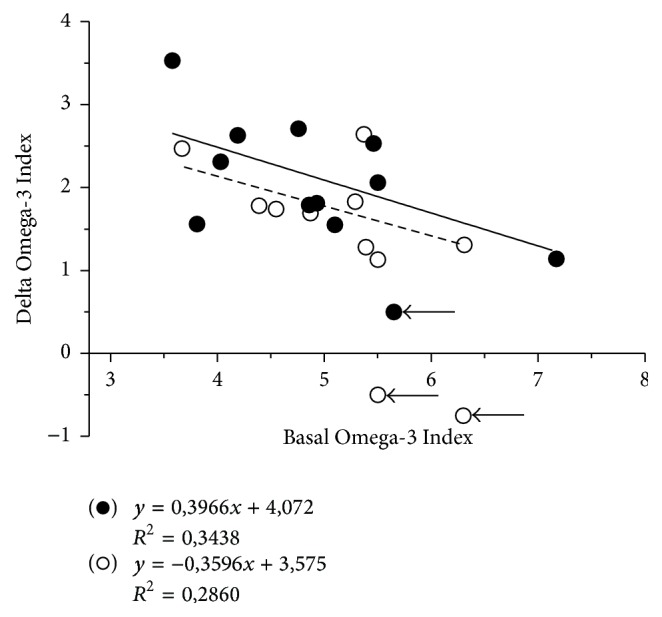
Changes on Omega-3 Index in athletes after 4 months of supplementation in function of the dose: (○) group 2G, two oil gums daily; (●) group 3G, three oil gums daily. The arrows show those subjects that were unable to comply with the treatment. These data were not added to the statistical evaluation; it is only to show the place in this figure. The line of tendency reflects the delta change of all subjects depending on the basal level and different doses.

A total of 41 individuals were screened between May and June 2014; twenty-four of them met the inclusion criteria and were randomly assigned to any treatment group. Besides, seven subjects were selected as control subjects. Since the study is to determine the level of impregnation in the tissue not a placebo but a control that maintained the same diet was considered, all baseline measurements were completed during the selection process. One subject withdrew from the study between baseline and the final point due to an injury during training, and he voluntarily dropped out from the study. In addition, two subjects from the 2G and one from the 3G groups comment the lack of compliance at the first control and desired to abandon the study. Among study completers, compliance was presumably total in all groups. Anyway, real compliance was assessed by interrogation, by counting returned capsules, and by analysis of red cell phospholipid fatty acid composition, which reflects dietary fatty acid composition. Those athletes, whose percentage of EPA in red cell membrane fatty acids differed, ≥2 Standard Deviations from the mean of the respective treatment group, were also considered noncompliant [20]. Noncompliant patients were excluded from the valid case analysis, but RBC analysis was performed confirming the absence of increase of EPA and consequently in O3Ix (Figure 1). Finally, from the 24 volunteers included in groups 2G and 3G, and once applied compliance criteria, the sample was reduced to nine and eleven subjects in both groups, respectively. Adherence to the therapy was 82% for 2G and 92% for 3G groups.

No significant differences were found between groups of participants with respect to baseline and diet characteristics. Nevertheless, the control group presented some differences related to age, height, and weight and slightly to the caloric intake, basically referred to as the technical staff. RBC FA content was similar between groups (Table 2). The mean O3Ix at study entry (mean ± SD) was 5.1 ± 1.0% with a range of 3.3% to 7.8%. On average, there were no gender differences in relation to O3Ix. Body weight, BMI, blood pressure, and heart rate did not change significantly during the study.

**Figure 2:**
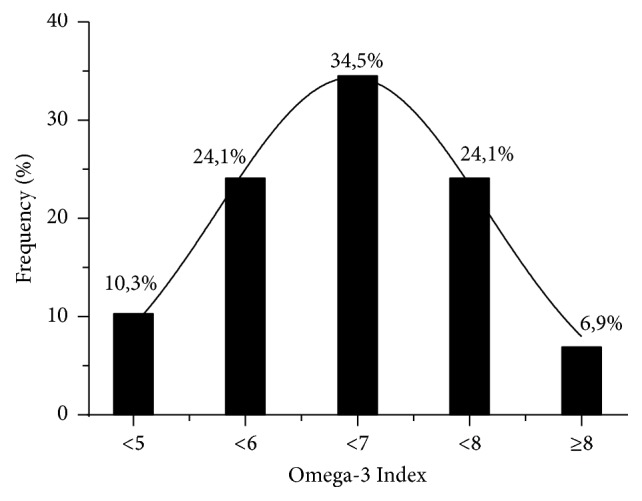
Distribution of the basal Omega-3 Index in the population of athletes of this study.

Distribution of O3Ix shows a Gauss distribution where a 93.1% of the athletes had values lower than 8% after EPA + DHA supplementation (Figure 2). The EPA + DHA supplementation increased the O3Ix in a dose-dependent manner (Table 2), affecting both EPA and DHA which resulted in a significant increase in O3Ix of 144% (116–157%) for the 3G dose and 135% (120–149%) for the 2G dose (*p* < 0.001). Participants who had lower basal O3Ix were more prone to increment their levels (Figure 1). Omega-3 FAs increase was accompanied by a significant decrease in total Omega-6 (n-6) FAs in both intervention groups from 28.0 to 25.2% (2G, *p* < 0.01) and 28.6 to 25.4% (3G, *p* < 0,001), respectively. No change in O3Ix was observed for the control group from baseline (93%). No significance changes were observed on the other FA except for MUFA, in the 3G group (20.0 ± 0.9 to 21.0 ± 1.0%).

## 4. Discussion

The present study evaluated the effect of EPA + DHA supplementation in athletes on the O3Ix response. The required amount of Omega-3 intake is not clearly defined, although there are certain recommendations based on individualized dietary patterns by state and age [21]. The World Health Organization establishes a need for consumption of 250–500 mg/day of EPA + DHA [22], while the International Society for the Study of Fatty Acids and Lipids [23] adjusts it to 500 mg/day. Certain effects as cardioprotective [24] or triglyceride decrease [25] at doses of 1 to 3–5 g/day of EPA + DHA are advised. In sport activities, similar dose also has been recommended [26]. However, the actual consumption of fatty fish does not become desirable for the different population groups and, as a result, the Omega-3 daily intake from the diet is insufficient. In Spain, the consumption of Omega-3 is 1.5 g/day with an average consumption of 0.2–2 g/day and it is estimated that Americans consume <100 mg/day of EPA + DHA [27]. While it may be considered satisfactory, the type of n-3 FAs consumed is basically ALA. As they have not the same biological effects and conversion of ALA to EPA and DHA is not carried out effectively, the EPA and DHA ingested are well below the recommendations of 0.25 to 0.5% of the daily energy, only reaching 0.05%. Moreover, the Omega-6/Omega-3 ratio in the same population is 15-16/1, well above the 4-5/1 that is considered suitable [28]. Therefore, the need for supplementation with n-3 FAs is real in all population age ranging from children to the elderly, and athletes are not out of this population.

We have found an average baseline O3Ix of 5.0% in the participants of the present study; this level is consistent with previous studies of adults' subjects reporting to be low habitual fish consumers [29] and in the same range of the athletes evaluated by Von Schacky et al. [13]. Our results suggest that athletes, with low fish intake who increased their dietary intake by 760 to 1140 mg/day of EPA + DHA, would experience an increase in O3Ix values of about 1.8% to 2.1% by mean, lower results than those observed when period of treatment is longer [30–32] over 3.5% and near to 5% increase (see Supplementary Material available online at https://doi.org/10.1155/2017/1472719). From these, it can be estimated that an average healthy adult with a low O3Ix (i.e., 5,0%) would require 1.5–2 g/day of EPA + DHA for 4 months or more to increase 2 index points and bring it to the desired 8%. Our different response could be related to the different ratio of EPA/DHA (44% EPA/56% DHA) as it has been argued by the different velocity of incorporation depending on that percentage [33]. That asseveration is not in agreement with Browning et al. study [30], with similar relation between DHA/EPA and fish oil content. Under this perspective, more concern has to be considered with the amount of Omega-3 offered and treatment duration, considering the different quantity and quality of products, which present different bioavailability [34].

**Figure 3:**
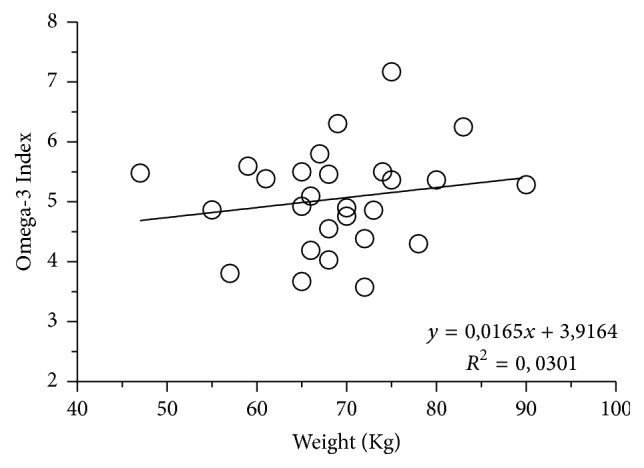
Relation of Omega-3 Index with body weight. There is a low correlation of the O3Ix distribution related to body weight in the population of this study.

**Figure 4:**
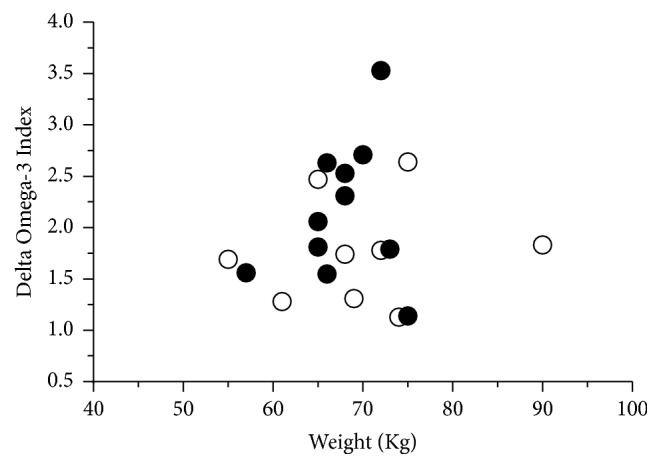
Delta change of the Omega-3 Index related to body weight and dosage: (○) group 2G, two oil gums daily; (●) group 3G, three oil gums daily. The changes on values of the O3Ix are more related to dosage than the previous body weight.

Body weight does not explain the variability of O3Ix in response to EPA + DHA supplementation in our data (Figures 3 and 4). Flock et al. [15] demonstrated a greater tendency to respond to a given EPA + DHA intake in individuals with lower body weight, suggesting that, to achieve a target O3Ix, consumption recommendations of EPA + DHA should be made on the basis of body weight, in a similar way as it happens with current dietary protein requirements. This discrepancy with our data could be due to the fact that the weight of the athletes is usually adequate to their physical activity needs and their caloric and nutrient intake, in all cases under their daily requirements (except for PUFA) [35]. In Folk et al.'s study [15], it was estimated that the requirements to increase its O3Ix from 4.3% to 8% in individuals weighing 75 kg were about 1.2 g/day of EPA + DHA; 0.9 g/day if they weighed 55 kg; or 1.5 g/day for individuals weighing 95 kg. As it can be observed in Figures 3 and 4, that observation does not correlate similarly in the subjects of our intervention. It seems that this adjustment of doses is not needed in the athletes if their weight is the expected for performance and nourishment is adequate. Possibly in athletes a higher dose needs to be administrated to achieve the desired 8% in O3Ix.

We also found that the EPA + DHA incorporation into the membranes of RBC follows a dose-dependent increase in both groups assayed, which is potentially saturable. This suggests that EPA + DHA concentrations in the membrane of RBC could be regulated to some degree and it could reach a saturation point. This finding is consistent with previous observation that individuals, with higher content of EPA + DHA in their RBC membranes, incorporate, at a slower rate, additional EPA + DHA than those presenting a lower baseline level [36].

We did not find that women on average had a higher O3Ix than men at study entry. This relationship between sex and O3Ix seems not to be related only to the difference in body weight [37] as it appears when this factor was accounted for in the model by adjusting the dose per unit of body weight [38]. In the same study, the participants, that are more physically active, tended to experience greater elevation in O3Ix as dose increased, suggesting that something related to exercise may enhance incorporation of EPA + DHA in RBC membranes in individuals taking fish oil supplements.

With respect to the MUFA change in the 3G group, with lower levels from the beginning, it can be attributed to the 334 mg of MUFA ingested daily, but it was not statistically different. MUFA consumption can be beneficial when replacing carbohydrate and saturated fat in the diet but not when replacing PUFAs. Although MUFA showed to have positive impact on surrogate markers, the potential impact of MUFA intake alone on disease outcomes, such as CVD or diabetes, remains unclear. Therefore, the role of MUFAs on health and disease when consumed as an eating pattern (i.e., Mediterranean diet) should be more studied [39]. Maybe the explanation of the favorable properties lies in the oleic acid, the MUFA most abundant fatty acid found in food, or, more particularly, in its minor though highly bioactive molecules, the phenolic compounds, which have been associated with the prevention of the main chronic diseases [40].

The results presented here show that elite athletes, despite following a diet presumably healthy and suitable for their sporting activity, have an O3Ix well below that recommended and that, despite a strict follow-up of the DHA + EPA supplement in the diet during the study period, they do not reach the desired levels. Given the potential role of DHA and EPA in cardiovascular protection, preservation of the central nervous system, repair of the musculoskeletal system, and significant influence on cellular behavior and responsiveness to signals [41], it would be advisable to increase the intake of these Omega-3 FAs in the diet of these athletes. A study of its long-term benefits is guaranteed.

## 5. Strengths and Limitations

The strengths of this study were as follows: sample of subjects with elevated physical activity who were under a controlled and healthy diet, single blind study design that compared two doses of EPA + DHA with respect to a control group with a high adherence to the treatment, adequate duration of supplementation, 4 months, and the use of validated analytical methods to determine biomarker response to treatment.

Limitations include the scarce sample, the homogeneity of white, young, and healthy population, and the lack of background genetic data.

## 6. Conclusions and Further Research

It is confirmed that athletes even with a presumably healthy diet have low O3Ix. The marine-derived n-3 FA supplementation increases the RBC EPA + DHA content in a dose-time related manner. Future studies need to assess how EPA or DHA individually or different ratios of both affect O3Ix response and to clarify the potential correlation between changes in the O3Ix and its effect on prevalence and severity of the injury recovery.

## Supplementary Material

Supplementary Data Table explores the comparison of different studies on DHA/EPA intake supplementation with respect to the present study in athletes.

## Figures and Tables

**Table 1:** Fatty acid composition of the diet supplement (gums).

Fatty acid	Composition^*∗*^ (%)	1 gum (mg)	2 gums (mg)	3 gums (mg)
*MUFA*				
Oleic (18:1, n-9)	18,5	111	222	334
Vaccenic acid (18:1, n-7)	3,1	19	37	56
Other MUFA	1,4	8	17	25

*SFA*				
Stearic acid (18:0)	1,4	8	17	25
Palmitic acid (16:0)	1,2	7	14	22
Other SFA	0,5	3	6	9

*PUFA n-6*				
Arachidonic acid (20:4, n-6)	1,7	10	20	30
Other n-6	1,9	12	23	35

*PUFA n-3*				
DHA (22:6, n-3)	35,7	214	428	643
EPA (20:5, n-3)	27,7	166	332	499
DPA (22:5, n-3)	3,3	20	40	60
Other n-3	3,5	21	42	63
Total DHA + EPA		380	761	1141

SFA, saturated fatty acid; MUFA, monounsaturated fatty acid; PUFA, polyunsaturated fatty acid; n-6, Omega-6; n-3, Omega-3; DHA, docosahexaenoic acid, EPA, eicosapentaenoic acid; DPA, docosapentaenoic acid. ^*∗*^To determine the quantitative fatty acid (FA) composition, FAs were analyzed by gas chromatography-mass spectrometry. The results express in molar % of total fatty acids.

**Table 2:** Erythrocyte fatty acids profile of the participants as a function of the dose through the study.

Mean (SD)	2G (N:9)			3G (N:11)			Control (N:6)			Control versus 2G/3G	
	Basal	Post	*p*≤	Basal	Post	*p*≤	Basal	Post	*p*≤	Basal	Post
SFA	44.7 (1.1)	45.6 (1.5)	*NS*	45.2 (0.7)	45.8 (0.8)	*0.05*	45.4 (0.9)	45.8 (0.8)	*NS*	*NS*	*NS*
MUFA	20.9 (1.6)	21.3 (1.2)	*NS*	20.0 (0.9)	21.0 (1,0)	*0.05*	21.0 (1.8)	21.9 (1.8)	*NS*	*NS*	*NS*
PUFA	34.4 (1.5)	33.6 (1.5)	*NS*	34.8 (0.9)	34.0 (1.2)	*0.05*	33.5 (1.4)	32.3 (1.4)	*0.05*	*NS*	*0,05*
n-6	27.9 (1.8)	25.2 (1.4)	*0.01*	28.6 (1.4)	25.4 (1.6)	*0.001*	27.5 (1.6)	26.7 (1.2)	*NS*	*NS*	*0,05*
n-3	6.4 (1.1)	8.4 (0.9)	*0.001*	6.3 (1.2)	8.6 (1.0)	*0.001*	6.1 (0.9)	5.6 (1.0)	*NS*	*NS*	*0,001*
n-6/n-3	4.5 (1.1)	3.0 (0.4)	*0.01*	4.7 (1.0)	3.0 (0.5)	*0.001*	4.6 (1.0)	4.9 (1.1)	*NS*	*NS*	*0,01*
O3Ix	5.0 (0.8)	6.8 (0.7)	*0.001*	4.9 (1,0)	7.0 (0,8)	*0.001*	4.8 (0,9)	4.4 (0,9)	*NS*	*NS*	*0,001*

Group 2G, two oil gums daily; 3G, three oil gums daily. SFA, saturated fatty acid; MUFA, monounsaturated fatty acid; PUFA, polyunsaturated fatty acid; n-6, Omega-6; n-3, Omega-3; and O3Ix, Omega-3 Index.
