# Supplementary material for: Erythrocyte Omega-3 Fatty Acid Content in Elite Athletes in Response to Omega-3 Supplementation: A Dose-Response Pilot Study
Source: J Lipids. 2017 Jun 1;2017:1472719. doi: 10.1155/2017/1472719 (PMC5471584; doi:10.1155/2017/1472719)
Supplement: Supplementary file 1 — Supplementary Data Table explores the comparison of different studies on DHA/EPA intake supplementation with respect to the present study in athletes. [file 1472719.f1.pdf]

## SUPPLEMENTARY DATA

Supplementary Data: Comparison of different studies on supplementation diet with DHA/EPA intake, respect the present study in athletes.

| Study (Reference) | %DHA/EPA vs Total FA | % DHA/EPA Ratio | DHA (mg/day) | EPA (mg/day) | Total DHA+EPA (mg/day) | Period (months) | Increment O3Ix (%) |
|---|---|---|---|---|---|---|---|
| Flock (29) | 13/20 | 40/60 | 720 | 1080 | 1.800 | 5 | 5.2 |
| Katan (32) | 28/6 | 15/85 | 300 | 1700 | 2.000 | 12 | 4 |
| Von Schacky (25) | 21/34 | 38/62 | 1140 | 1860 | 3.000 | 3 | 5.5 |
| | | | 380 | 620 | 1.000 | 12 | |
| Browning (47) | 39/33 | 54/46 | 1026 | 874 | 1.900 | 12 | 5 |
| Tartibian (49) | 12/18 | 40/60 | 400 | 600 | 1.000 | 3 | 5.1* |
| Hill (48) | 26/6 | 81/19 | 1540 | 360 | 1.900 | 4 | 3.5** |
| Present study | 36/28 | 56/44 | 426 | 334 | 760 | 4 | 1.4 |
| | | | 638 | 502 | 1.140 | 4 | 1.9 |

*Cells studied were neutrophils. **The result is the sum of DHA+EPA+DPA.
